# Novel Approach Combining Transcriptional and Evolutionary Signatures to Identify New Multiciliation Genes

**DOI:** 10.3390/genes12091452

**Published:** 2021-09-21

**Authors:** Audrey Defosset, Dorine Merlat, Laetitia Poidevin, Yannis Nevers, Arnaud Kress, Olivier Poch, Odile Lecompte

**Affiliations:** 1 Department of Computer Science, ICube, UMR 7357, Centre de Recherche en Biomédecine de Strasbourg, University of Strasbourg, CNRS, 67000 Strasbourg, France; adefosset@etu.unistra.fr (A.D.); dorine.merlat@etu.unistra.fr (D.M.); l.poidevin@unistra.fr (L.P.); akress@unistra.fr (A.K.); olivier.poch@unistra.fr (O.P.); 2 Department of Computational Biology, University of Lausanne, 1015 Lausanne, Switzerland; yannis.nevers@unil.ch; 3 Center for Integrative Genomics, University of Lausanne, 1015 Lausanne, Switzerland; 4 Swiss Institute for Bioinformatics, University of Lausanne, 1015 Lausanne, Switzerland

**Keywords:** multiciliation, rare diseases, comparative genomics, evolution, functional genomics, multi-omics data integration

## Abstract

Multiciliogenesis is a complex process that allows the generation of hundreds of motile cilia on the surface of specialized cells, to create fluid flow across epithelial surfaces. Dysfunction of human multiciliated cells is associated with diseases of the brain, airway and reproductive tracts. Despite recent efforts to characterize the transcriptional events responsible for the differentiation of multiciliated cells, a lot of actors remain to be identified. In this work, we capitalize on the ever-growing quantity of high-throughput data to search for new candidate genes involved in multiciliation. After performing a large-scale screening using 10 transcriptomics datasets dedicated to multiciliation, we established a specific evolutionary signature involving Otomorpha fish to use as a criterion to select the most likely targets. Combining both approaches highlighted a list of 114 potential multiciliated candidates. We characterized these genes first by generating protein interaction networks, which showed various clusters of ciliated and multiciliated genes, and then by computing phylogenetic profiles. In the end, we selected 11 poorly characterized genes that seem like particularly promising multiciliated candidates. By combining functional and comparative genomics methods, we developed a novel type of approach to study biological processes and identify new promising candidates linked to that process.

## 1. Introduction

Cilia are ancient and complex organelles found on the surface of most eukaryotic cells, in the form of a single non-motile projection used as a sensory device capable of transducing signals during development and homeostasis (reviewed in [1]). Some specialized cells, however, can generate several dozens of motile cilia on their surface that beat in a coordinated manner to generate a directional fluid flow used to circulate liquids or give movement to particles [2]. In mammals, particularly in humans, multiciliated cells (MCCs) are found in the spinal cord and the brain ventricles, where they direct cerebrospinal fluid flow in the respiratory tract and play an important role in mucus clearance, as well as in both male and female reproductive tracts (reviewed in [3]). MCCs have also frequently been studied in other vertebrate species, such as in *Xenopus laevis* tadpoles that possess a mucociliary epidermis [4], and in *Danio rerio*, where they can be found in the pronephros [5]. 

**Figure 1:**
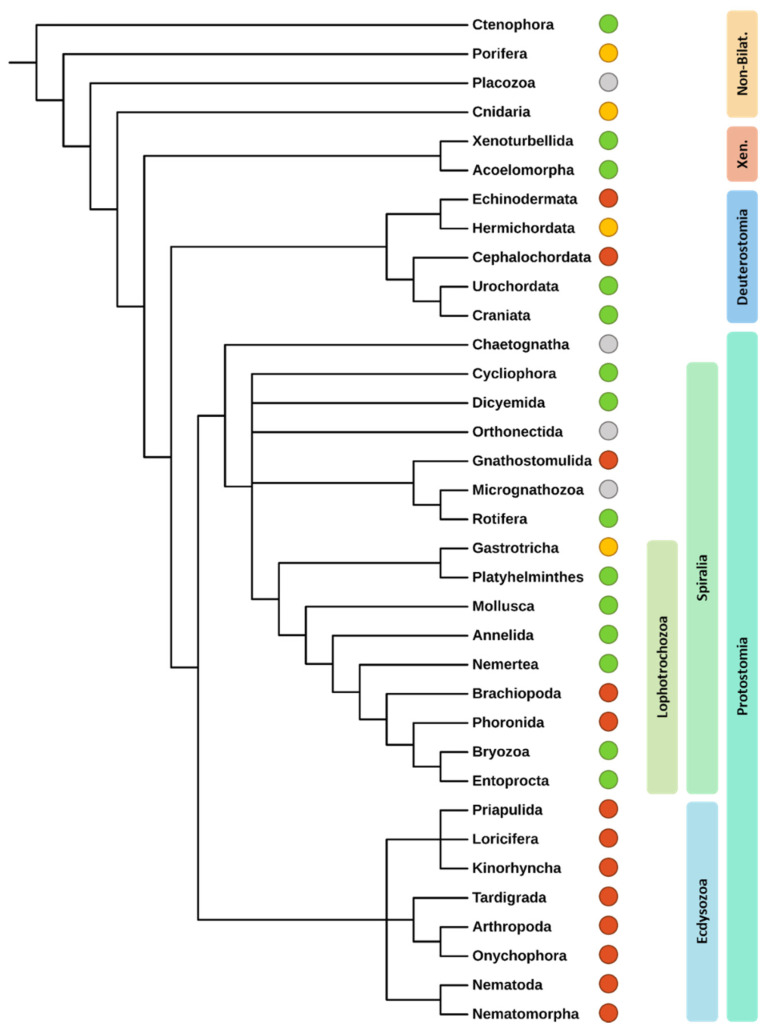
Distribution of MCCs in different metazoan clades. A green circle indicates the presence of MCCs, a red circle indicates their absence. A yellow circle indicates the existence of both multiciliated species and strictly monociliated species in the same branch. Gray circles indicate a lack of information regarding multiciliation. Xen.: xenacoelomorpha; Non-Bilat: non-bilaterian. Phylogeny based on the results of Giribet [12].

Multiciliation is a process found throughout evolution, but its exact presence or absence in all eukaryotic clades has not yet been clearly established. Multiple accounts of MCCs in Metazoa have been given in the literature, although some unknowns remain; they have been observed in non-bilaterian species, in some Protostomes but not in others, with a clear absence in Ecdysozoa, as well as in most subphyla of Deuterostomia [6] (Figure 1). While MCCs have been observed in various unicellular species such as *Multicilia marina*, *Tetrahymena* and *Paramecium* [7,8,9] as well as in sperm of certain plants [10,11], it is unclear whether the mechanisms responsible for the generation of multiple motile cilia are the same, though it seems unlikely. 

**Figure 2:**
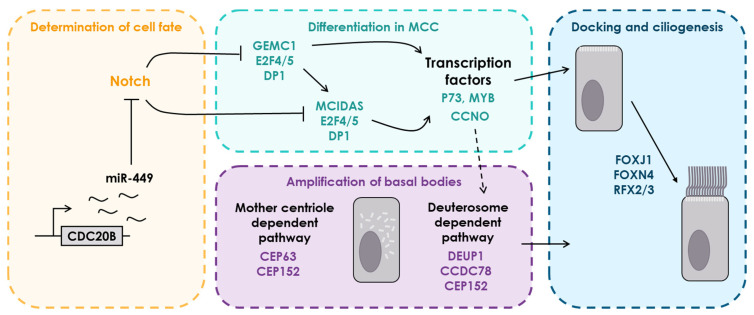
Schematic representation of the main stages of multiciliogenesis, with major genes. Flat-ended arrows depict the repression of a gene, regular arrows depict the activation of a gene.

Over the years, studies have allowed us to get a better understanding of the transcriptional cascade involved in the differentiation of MCCs in vertebrates (Figure 2, reviewed in [13]). The roles of the Notch signaling pathway are numerous during development; in multiciliated epithelia, various studies have shown its function as a determinant of the cellular fate, either into a secretory cell in the presence of Notch, or into a multiciliated cell in its absence [14,15,16]. One of the earliest steps of the multiciliated pathway identified involves a family of micro-RNAs, the *miR-34/449* family, which contains six members in mammals, three of which are hosted by the same gene, *CDC20B*. The role of these miRNAs is to promote the exit from the cell cycle and inhibit Notch1 receptors, thus provoking the sealing of the multiciliated fate in the cell [17]. STK11, a serine/threonine kinase, has recently been identified as a necessary actor to permit differentiation of MCCs, presumably by inhibiting cell proliferation through a signaling cascade involving MARK3, ERK1/2, although its relation with other multiciliated determinants remains to be clarified [18].

Once the multiciliated fate has been determined, the inhibition of *Notch* allows the activation of the two central mediators of MCCs differentiation: GEMC1, encoded by *GMNC*, and multicilin/MCIDAS, encoded by *MCIDAS*, members of the geminin protein family, characterized by the presence of coiled-coil domains [19,20,21,22]. Both indispensable for the development of MCCs, GEMC1 and MCIDAS bind to E2F4/5 and DP1 to activate various transcription factors necessary for the steps leading to the generation of multiple motile cilia [22,23]. Among MCIDAS and GEMC1 targets are *p73* and *MYB*, two other important regulators of multiciliation also known to activate transcription factors of the multiciliated pathway [24,25,26]. 

One of the most important and challenging aspects of multiciliogenesis is the efficient generation and docking of several dozens of basal bodies (BB) from which cilia will later develop. So far, two pathways have been identified in BB amplification: the mother centriole dependent (MCD) pathway, and the deuterosome dependent (DD) pathway [27]. However, this step of multiciliogenesis remains blurry; it was previously thought that the DD pathway accounted for up to 95% of BBs, but recent studies have shown that the absence of either of both amplification pathways did not prevent the generation of procentrioles [28,29]. 

The canonical MCD pathway depends on two centrosomal proteins, CEP63 and CEP152, to form a ring like structure around the mother centriole from which new centrioles will then stem. The DD pathway relies on CEP63 paralog DEUP1 and CEP152 for de novo BB generation, through a ring-shaped structure called the deuterosome. CEP63 and DEUP1 both associate with CEP152 in a mutually exclusive manner, and in *X. laevis* it was shown that DEUP1 requires the presence of CCDC78 to recruit Cep152 and form new centrioles [30]. To allow for the adequate formation of deuterosomes in terms of number, structure and shape, CCNO, an MDICAS target, is also required, although its exact role remains to be determined [31].

Essential to BB migration, docking and ciliogenesis, FOXJ1 is one of the first transcription factors identified as necessary to the development of MCCs, notably through its large number of target genes [32]. Since then, several studies have shown that its activity could be influenced by RFX2 and RFX3, and that FOXN4 shares a non-negligible number of targets with FOXJ1. Together, these four transcription factors control the expression of numerous genes involved in BB docking and ciliogenesis. 

Despite the growing number of works dedicated to the study of multiciliation, quite a few gray areas remain regarding the transcriptional events responsible for the differentiation of MCCs. So far, only a few genes have been associated with anomalies of multiciliation, responsible of pathologies characterized by chronic respiratory infections from a young age, hydrocephalus and, in all likelihood, sterility [33]. As of yet, only MCIDAS [34], CCNO [35] and FOXJ1 [36] have been identified as causative of reduced generation of motile cilia (RGMC). 

To identify new candidate genes involved in multiciliogenesis, we capitalized on the ever-growing quantity of biological data available to realize a large-scale screening of potential genes using several genomics techniques. We analyzed ten transcriptomics datasets of experiments dedicated to multiciliation to look for genes overexpressed in MCCs and combined our results with comparative genomics in an unprecedented study. We identified a specific evolutionary pattern of multiciliation involving a group of bony fish, the Otomorpha (*D. rerio, Astyanax mexicanus*, etc.), that allowed us to prioritize target genes that were brought out in our transcriptomics analysis. The integration of both functional and comparative genomics allowed us to identify 11 new candidates that appear particularly promising. 

## 2. Materials and Methods

### 2.1. Functional Genomics Analysis

#### 2.1.1. Public Transcriptomic Datasets

Eight datasets comprised of results from ten transcriptomics experiments specific to multiciliation (Table 1) were retrieved from the Gene Expression Omnibus (GEO) database of the NCBI [37] for our functional genomics analysis. The selected datasets are either microarray or RNAseq experiments conducted on *X. laevis* or *Mus musculus* and consist in the inactivation of genes involved in the currently known multiciliation pathway. 

GSE32452. In this experiment, Stubbs et al. compare gene expression between *X. laevis* embryo in which Notch intracellular domain (ICD) was injected and embryos in which both ICD and a glucocorticoid inducible multicilin were injected [19]. 

GSE59309. In this experiment, authors compare gene expression in epithelial progenitors of *Xenopus* embryos in which multiciliation was induced by multicilin either with wild type E2F4 or a truncated form of E2F4 missing the last 140 amino acids [23].

GSE89271. This dataset includes data from 3 experiments aimed at the characterization of *Foxn4* and its role in multiciliation compared to *Foxj1*. Thus, gene expression of control embryos with glucocorticoid inducible multicilin was compared with three states: (1) embryos injected with glucocorticoid inducible multicilin and *Foxn4* morpholino, (2) embryos injected with glucocorticoid inducible multicilin and CRISPR/Cas9 system aimed at *Foxn4* or (3) embryos injected with glucocorticoid inducible multicilin and CRISPR/Cas9 aimed at *Foxj1* [38]. 

GSE76342. Quigley and Kintner compare gene expression in epithelial progenitors of *Xenopus* at three timepoints (3, 6 and 9 h) in three pairs of conditions: (1) Notch blocking compared to injection of ICD, (2) injection of ICD compared to injection of ICD and multicilin and (3) *Notch* blocking compared to *Notch* blocking with inactive multicilin [39]. 

GSE60365. In this experiment, authors compare gene expression in mouse tracheal cells when injected with control shRNA or with *Myb* blocking shRNA [40]. 

GSE75715. Authors compare wild type individuals with *p73* knockout mice [25].

GSE73331. Mori et al. compare wild type mice with *E2F4* knockout mice [41].

GSE116690. In this dataset, authors compare gene expression in lung cells of wild type mouse with cells of mice in which *STK11* was deleted in lung progenitors using the Cre-lox system [18]. 

#### 2.1.2. Raw Data Analysis

When available, the overexpression results provided by the authors were used directly (GSE89271, GSE76342, GSE78715 and GSE116690), otherwise, raw data were analyzed with the GEO2R tool of the NCBI [37] (GSE32452, GSE60365, GSE73331) and genes significantly overexpressed in a multiciliated state were retrieved. In the case of the GSE59309 dataset, the raw data were not directly analyzable with GEO2R, we thus followed the same method the authors describe in the manuscript and used the DESeq2 R package [42] to analyze each time point individually. For all datasets, the logFC threshold was set as ≥1 and as ≤0.05 for the *p*-value. 

#### 2.1.3. Overexpressed Genes Clustering 

Before comparing overexpressed genes of the different datasets previously cited, we had to homogenize sequences identifiers, as the datasets come from different platforms and species. We thus used orthologous human gene names retrieved with the Mouse Genome Informatics database (http://www.informatics.jax.org/ (accessed on 26 November 2020)) and Xenbase [43] for mouse and xenopus sequences respectively. 

We generated a binary matrix with the results of the 10 experiments indicating the presence (1) or the absence (0) of overexpression for each gene (as rows) in each experiment (as columns). Pairwise distance between gene profiles was calculated using the *dist* function of the *amap* R package, using the ‘binary’ method (https://CRAN.R-project.org/package=amap (accessed on 2 December 2020)). Hierarchical clustering was then carried out using the *hclust* R function with the ‘ward.D2’ method. Finally, gene clusters were defined through dynamic trimming of the resulting dendrogram using the *cuttreeDynamic* function of the *dynamicTreeCut* R package (https://CRAN.R-project.org/package=dynamicTreeCut (accessed on 4 December 2020)) using a depth of 2 and the ‘hybrid’ cut method. 

### 2.2. Comparative Genomics Analysis

#### 2.2.1. Evolutionary Analysis of Multiciliated Genes 

For the evolutionary study of known multiciliated genes, we used the NCBI implementation of BLASTp [44] with default parameters to search for orthologous sequences, using the human sequence as a query. When necessary, gene absence was confirmed using tBLASTn on the latest release of the relevant species’ genome, with default parameters and a word length of 3. Multiple sequence alignments of proteins were computed using the PipeAlign2 tool (http://www.lbgi.fr/pipealign (accessed on 12 January 2021)) [45], using MAFFT as the main alignment software [46] and manually analyzed. Genomic context of selected genes was analyzed on the Ensembl platform [47] or the NCBI sequence viewer [48]. 

#### 2.2.2. Search for Atypically Conserved Genes in Otomorpha 

To search proteins with a marked sequence divergence in Otomorpha, we used BLUR [49], a tool designed by the authors with the purpose of comparing groups of species to identified unexpected variations in sequence. We compared Otomorpha fish with species of the clade Euteleosteomorpha, another group of bony fish. We used the human proteome as a reference and chose to work with orthologs. 

### 2.3. In Depth Analysis of Target Genes 

#### 2.3.1. Generation of Interaction Networks 

We used the STRING protein interaction database [50] to construct our interaction networks and retrieved all genes that had interactions with at least 2 genes of our target list. We used a threshold score of 0.7 for interactions between the genes of our list, and 0.9 for interactions between genes of our list and external genes. We used Cytoscape [51] in combination with the GLay community clustering algorithm [52] implementation in the clusterMaker2 Cytoscape plugin [53]. 

#### 2.3.2. Computation and Clustering of Phylogenetic Profiles

OrthoInspector 3.0 [54] was used to retrieve orthologs of our target genes in 711 eukaryotic species, with which we generated a matrix containing the presence or absence of each gene in those species. Once the phylogenetic profiles were constructed, we used the *correlation* method of the *amap* R-package. Hierarchical clustering was then carried out using the *hclust* R function with the ‘ward.D2’ method and trimming was done using the *cuttreeDynamic* function of the *dynamicTreeCut* R package with a depth of 2 and the ‘hybrid’ cut method (see Section 2.1.3). 

## 3. Results

To expand our current knowledge regarding the generation and maintenance of MCCs, we combined functional genomics and comparative genomics data in order to highlight genes that were likely to be involved in this process. We first looked at expression data of multiciliation oriented transcriptomics data, then used specific evolutionary patterns to pinpoint the most probable target genes. 

### 3.1. Functional Analysis of Multiciliation Oriented Experiments

The first step of our study was the analysis of transcriptomics data from experiments designed to compare the multiciliated state and the non multiciliated state of cells. In this step, we looked for genes that were overexpressed in a multiciliated state in more than one experiment. 

#### 3.1.1. Gene Overexpression 

To identify genes overexpressed in MCCs, we selected 8 transcriptomics datasets comprising 10 experimental designs aimed at comparing MCCs to cells in which multiciliation was altered by inactivating genes known to be involved in the generation of MCCs (Table 1, see Section 2.1.1). These experiments can be grossly classified in two types depending on when the studied gene acts in the pathway: ‘early’ genes, responsible for the overall regulation of multiciliation (*STK11*, *p73*, *MCIDAS* and *E2F4*) and ‘late’ genes, responsible for the activation of multiple motile cilia generation (*Myb*, *Foxn4* and *Foxj1*). 

For each dataset, we retrieved genes overexpressed in multiciliated condition using a threshold of 1 for logFC and 0.05 for *p*-value, for a total of 4151 genes differentially expressed in at least one experiment. We compiled these results in a two-dimensional binary matrix with each gene as a row and each experiment as a column, and in each cell, the value 1 is entered if the gene is overexpressed, and 0 if it is not. 

#### 3.1.2. Gene Clustering

**Figure 3:**
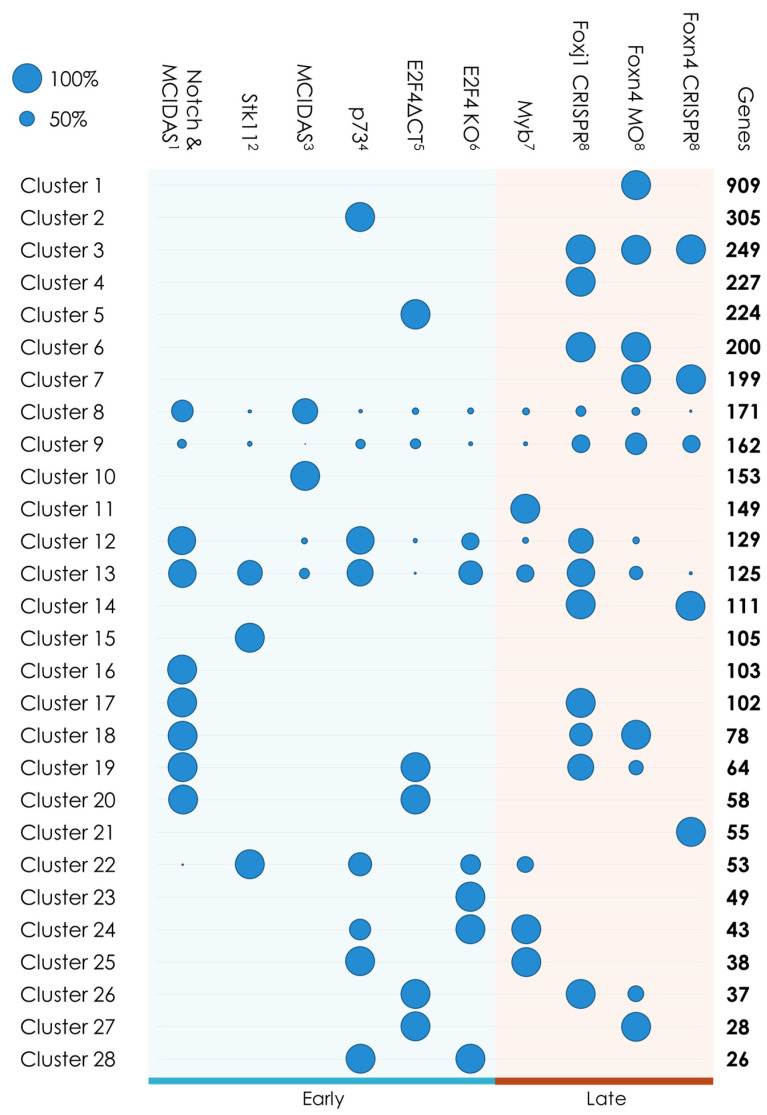
Schematic representation of the clustering of the results from the transcriptomics datasets. Circle size is proportional to the percentage of the cluster’s genes found in each dataset. ^1^GSE76342; ^2^GSE116690; ^3^GSE32452; ^4^GSE75715; ^5^GSE59309; ^6^GSE73331; ^7^GSE60365; ^8^GSE89271. ‘Early’ genes (*STK11*, *p73*, *MCIDAS* and *E2F4*) are responsible for the overall regulation of multiciliation. ‘Late’ genes (*Myb*, *Foxn4* and *Foxj1*) activate the generation of motile cilia.

To further highlight genes of interest, we carried out a clustering of the various expression results using the Jaccard index applied to each gene profile to measure the distance between them, as well as the Ward algorithm [55] to perform the clustering (see Section 2.1.3). We obtained 28 clusters with 10 containing genes overexpressed in only one experiment, for a total of 2278 genes that will not be taken in consideration for the rest of our study (Figure 3). 

The 18 remaining clusters can be regrouped according to the similarity of their expression profile and Panther [56] was used to perform Gene Ontology (GO) [57] term enrichments on each group of clusters to identify associated biological processes (Table 2).

Clusters 3, 6, 7 and 14 contain ‘late’ genes that seem to be under the influence of Foxn4 and Foxj1 whose roles, as we mentioned before, are largely redundant. GO term enrichments reveal an overrepresentation of genes involved in the establishment of the localization of biological components, which may both be linked to the numerous roles played by the FOX protein family as well as the large need for vesicular transport mechanisms during ciliogenesis [58]. 

Clusters 17, 18, 19, 26 and 27 hold genes influenced by both the MCIDAS/E2F4 complex and one or both of its targets, Foxn4 and Foxj1. These clusters are largely enriched in genes linked to cilia related GO terms such as ‘cilium organization’ (*p*-value: 8.28 × 10^−23^) or ‘cilium assembly’ (*p*-value: 1.26 × 10^−20^). Interestingly, 44 out of the 308 genes found in those clusters are not associated with any GO terms in *Homo sapiens* or *M. musculus*. 

Some clusters (22, 24, 25 and 28) contain genes influenced by a combination of STK11, p73, E2F4 and Myb, but more importantly, these genes only result from experiments done on the mouse. That can either be due to a specific regulation of multiciliation only found in mice, or to the absence of orthologs of these genes in *X. laevis*. 

Cluster 20 contains genes influenced only by a variation in the expression of *MCIDAS* and *E2F4* and that seem mostly involved in multiplication of basal bodies, with enrichment in GO terms such as ‘centriole replication’ (*p*-value: 1.02 × 10^−12^), ‘centriole assembly’ (*p*-value: 2.58 × 10^−12^) or ‘centrosome duplication’ (*p*-value: 1.22 × 10^−11^). 

Finally, the last clusters (8, 9, 12, 13) contain genes influenced by a large number of multiciliated factors and, as such, these clusters also contain the majority of genes known to be involved in this process. Thus, *MCIDAS*, *DEUP1*, *CEP152*, *PLK4*, *MYB*, *CCP110*, *CDC20B*, *FOXJ1* and *FOXN4* can be found in cluster 8, *CCNO* and *CCDC78* are in cluster 13, and *RFX2* and *RFX3* are in cluster 12. In addition to a strong enrichment in GO terms linked to cilia, these clusters contain 60 genes for which no GO annotation can be found in human orthologs. 

### 3.2. Comparative Genomics 

The first step of our study generated a large list of potential genes linked to multiciliation, however in most cases, the dissociation of ciliation and multiciliation is impossible. To overcome this limitation, we used comparative genomics to identify and exploit a specific evolutionary pattern of multiciliation found in some species of bony fish, the Otomorpha. 

#### 3.2.1. Identification of Evolutionary Pattern

**Figure 4:**
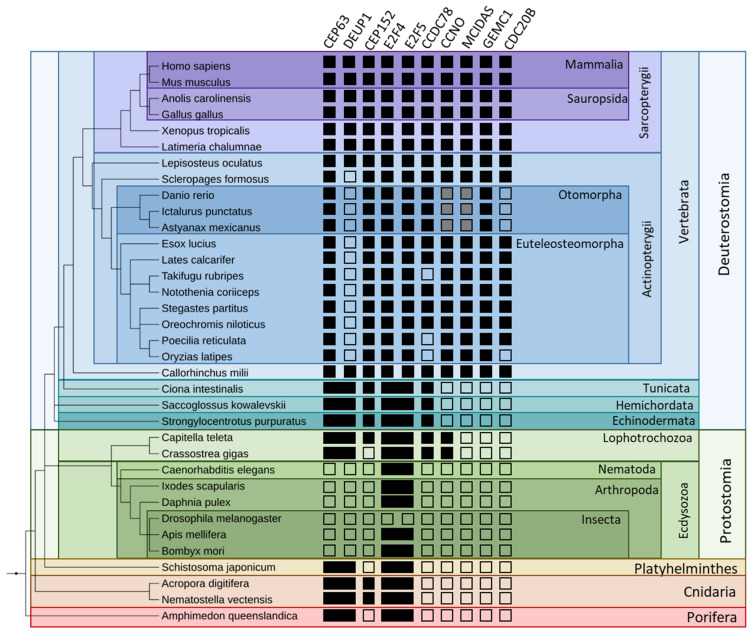
Phylogenetic distribution of multiciliated genes in Metazoa. Only a selected pertinent subset of the studied species is represented. A black square indicates the presence of an ortholog in the concerned species, a blank square indicates the absence of an ortholog. Black rectangles indicate the presence of an ancestral gene before its split in two paralogs. Gray squares indicate the presence of a homologue with an abormaly divergent sequence.

To begin our search for a multiciliation-specific evolutionary pattern, we identified orthologs of the following proteins in Metazoa using BlastP and human sequences as a query on the RefSeq protein database [59]: CEP63, DEUP1, MCIDAS, GEMC1, CCNO, CCDC78, E2F4, E2F5, CEP152 and CDC20B. We established the phylogenetic distribution of each gene in selected metazoan organisms to identify a phylogenetic profile linked to MCCs but most of the genes studied have different and seemingly complex evolutionary histories which did not enable us to construct a unique evolutionary signature (Figure 4). Some genes like *CCDC78, CCNO, MCIDAS, GMNC* and *CDC20B* seem to have appeared more recently and are mostly limited to Vertebrata, while the others seem to be older and present orthologs in most Metazoa. Interestingly, no orthologs were found in Ecdysozoa except for E2F4 and E2F5, which is consistent with the absence of MCCs in those species, and CDC20B is absent in a group of bony fish, the Otomorpha. These absences were verified at the genomic level by TBLASTN searches.

We then focused on the conservation of orthologous sequences through evolution at the subprotein level by analyzing protein multiple sequences alignments. While most of the proteins are relatively well conserved in all taxa studied, two proteins stand out with a particular sequence variation in specific bony fish species of the Otomorpha clade: MCIDAS, which was previously shown in zebrafish [60] and CCNO. 

**Figure 5:**
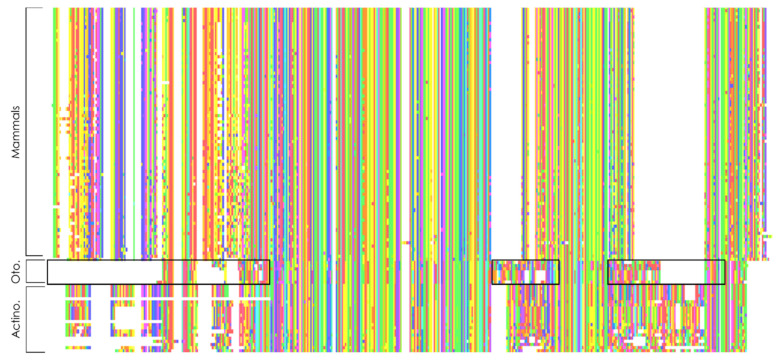
Overview of CCNO multiple sequence alignment. Framed areas show parts of the alignment with the most divergence in Otomorpha fish when compared to other species. Oto.: Otomorpha; Actino.: Actinopterygii.

In the case of CCNO, Otomorpha fish proteins present divergences along the whole sequence when compared to mammals or other bony fishes, with the N-terminal portion of the sequence truncated (Figure 5). As for MCIDAS, both the coiled-coil domain, used for interaction with GEMC1, Geminin and MCIDAS itself, and the TIRT domain, used for interaction with E2F4 and E2F5 [23] are relatively well conserved in most species, including Otomorpha. The rest of the protein sequence, however, is largely divergent in the latter species, with an almost absent N-terminal domain, while being overall similar between other bony fish species and Sarcopterygii. 

**Figure 6:**
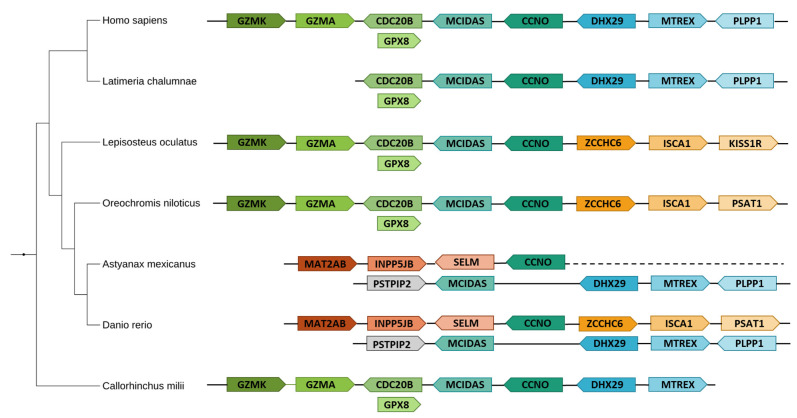
Multiciliated locus in selected vertebrate species. The locus contains *MCIDAS*, *CCNO* and *CDC20B*, three genes essential for multiciliation. This synteny block is conserved in most vertebrate species except in Otomorpha fish (*Danio rerio, Astyanax mexicanus*).

Lastly, we looked at the genomic localization of multiciliated genes, studying a well-known locus conserved in humans, mice and frogs containing *MCIDAS*, *CCNO* and *CDC20B* [19]. We focused our search on bony fish to see if this syntenic block could be seen beyond tetrapods and found that the co-localization of *MCIDAS*, *CCNO* and *CDC20B* exists in *Latimeria chalumnae*, certain species of Euteleosteomorpha fish, as well as in *Callorhinchus milii*, a cartilaginous fish. Interestingly, this gene co-localization is known to be broken in zebrafish [60], but our study showed that this extends to other Otomorpha fish, with *MCIDAS* on one chromosome and *CCNO* on another, and *CDC20B* gone entirely (Figure 6). 

All these results point towards complex evolutionary events that seem to have happened in Otomorpha fish that lead to the loss of *CDC20B* and the *mir-449* family, as well a possible sequence variation in CCNO and MCIDAS. 

#### 3.2.2. Multi-Level Identification of Differential Conservation

Our evolutionary study showed the existence of gene loss as well as protein sequence divergence in several of the key genes of multiciliation in the Otomorpha group of fish. Based on these results, we searched for other genes that would either be missing in Otomorpha while being present in other bony fish, or genes that would have diverged in an unexpected way in Otomorpha, in order to identify new multiciliated candidates. We used BLUR [49], a tool designed to detect differential conservation in specific species both at the protein and sub-protein level, and compared Otomorpha to Euteleosteomorpha using the human proteome as a reference. 

Out of the 21,044 proteins found in the human proteome, 1361 present an unexpected behavior in Otomorpha when compared to Euteleosteomorpha fish; 634 have no orthologs, 104 are highly likely to present a sequence divergence in Otomorpha, and 623 are mildly likely to present a divergence according to BLUR. Interestingly, MCIDAS is found among the highly likely targets. GO term enrichment of the 1361 proteins showed a slight overrepresentation of proteins linked to immunity, suggesting that more than one process might be divergent between Otomorpha and Euteleosteomorpha fish and thus making it important to cross these results with the ones stemming from our functional genomics analysis. 

### 3.3. Integration of Functional and Comparative Genomics Results 

With the aim of distinguishing genes linked to ciliation and genes specific to multiciliation, we used comparative genomics to identify a multiciliation-specific evolutionary pattern in the form of unexpected gene divergences in Otomorpha fish, to use as a filter on target genes found with our transcriptomics study. The integration of both approaches allowed us to generate a list of 114 genes (Supplementary Table S1) overexpressed in multiciliated conditions, that are either absent in Otomorpha fish or show an unexpected sequence divergence when compared to Euteleosteomorpha fish. Among the 114 targets, 41 are absent in Otomorpha, 10 are highly likely to be differentially conserved in Otomorpha and 63 are mildly likely to present an atypical sequence divergence in Otomorpha. 

To further analyze our target genes and have a comprehensive picture of multiciliation, we added eight known multiciliated genes to our list (*CCNO, GEMC1, CEP63, CEP152, E2F4, E3F5, CCDC78* and *CDC20B*) and used two characterization approaches: one using functional interaction networks and one using phylogenetic profiling. 

#### 3.3.1. Functional Interaction Networks

**Figure 7:**
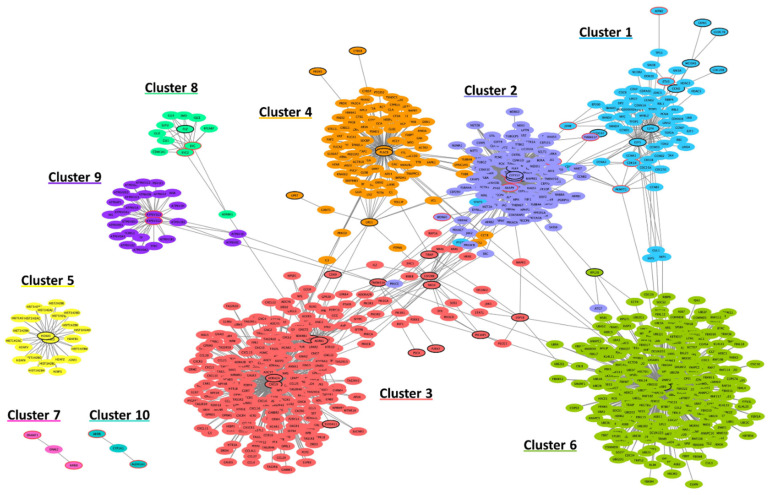
Interaction network computed from the list of 114 potential multiciliated targets as well as genes presenting a functional interaction with at least two genes of that list. These clusters contain 57 of our genes (circled in bold) and a total of 919 genes.

To characterize our target genes, we used functional interaction networks with the hypothesis that proteins that interact together tend to be involved in the same biological process. With this in mind, we broadened our gene selection to build a sizeable network that allowed us to identify functional clusters. We used the STRING protein interaction database to select additional proteins that interacted with at least two of the proteins of our 122 targets, thus creating a network of 919 genes, including 57 from our list, divided into 10 clusters (Figure 7). 

We performed GO enrichment analyses for each cluster and found that six out of ten clusters are either directly or indirectly linked to multiciliogenesis or, more broadly, to ciliogenesis. Cluster 1, while being enriched in proteins related to the regulation of the cell cycle (*p*-value: 1.53 × 10^−34^), contains eight proteins currently known to be involved in multiciliation (GEMC1, CCDC78, MCIDAS, CDC20B, CCNO, E2F4, E2F5 and DEUP1). Clusters 2 and 8 both show an enrichment in proteins localized to the centrosome (*p*-value: 2.75 × 10^−108^) and cilium (*p*-value: 7.26 × 10^−16^) respectively, and as such contains proteins that could be involved in ciliation as well as in multiciliation. Cluster 9 is enriched in GO terms related to autophagy (*p*-value: 4.22 × 10^−32^), a mechanism involved in centrosome number regulation [61]. Finally, clusters 7 and 10, although containing only 3 proteins each, are respectively enriched in the terms ‘cilium movement’ (*p*-value: 4.18 × 10^−7^) and ‘retinoid metabolic process’ (*p*-value: 1.87 × 10^−5^), the latter of which might seem unrelated to multiciliation, but recent studies have shown that retinoic acid is involved in the regulation of multiciliogenesis in *D. rerio* [62]. Those six clusters contain 32 of the 57 target genes, among which 20 are either of unknown function, or are currently unrelated to cilia (Supplementary Table S1). 

#### 3.3.2. Phylogenetic Profiling of Multiciliated Targets

We mentioned above that multiciliation is a complex process with an even more complex evolutionary history, with specific losses in some clades such as the Ecdysozoa and a fair number of species for which information is still lacking. With this is mind, we used phylogenetic profiling and clustering to identify genes amongst our targets with profiles that correlate the most with multiciliation. To permit a more comprehensive clustering and a better view of multiciliary evolution, we reused the genes of our previous interaction network analysis. 

**Figure 8:**
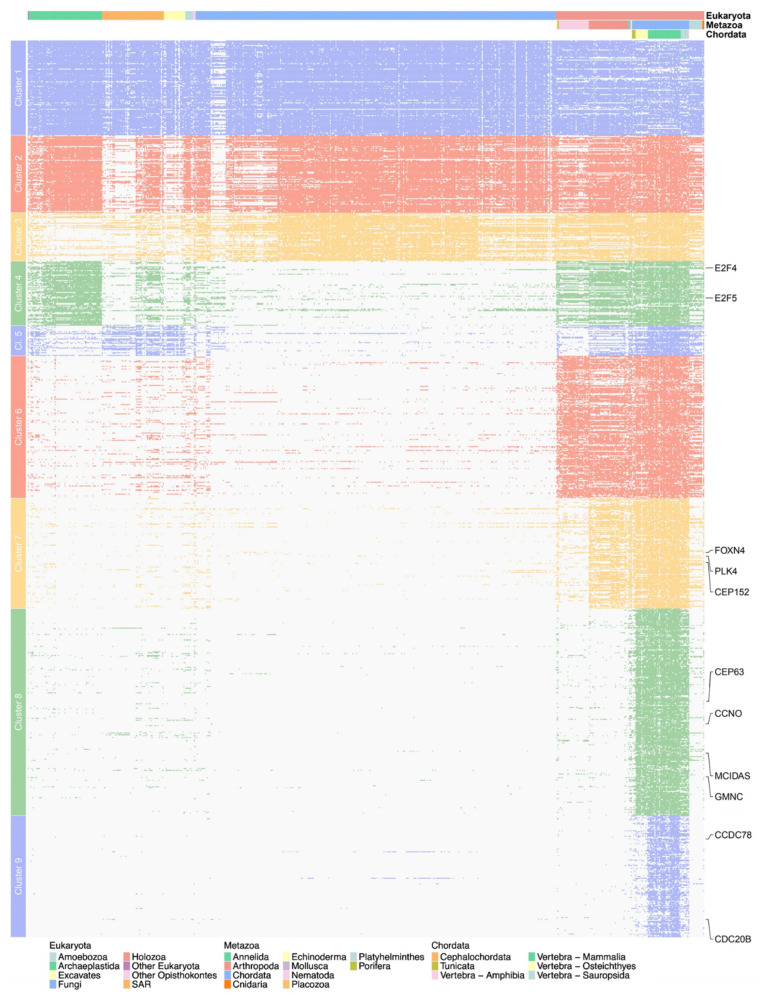
Evolutionary clustering of 984 human genes based on their phylogenetic distribution in 711 eukaryotic species. Rows represent genes and columns represent species, with each colored square indicating the presence of an ortholog of the concerned gene in the chosen species. The three lines above the profiles indicate the main clades in Eukaryota, Metazoa and Chordata respectively.

We used OrthoInspector to generate phylogenetic profiles for our 984 genes in 711 eukaryotic species and clustered them based on the distance between the profiles (Figure 8). Nine clusters were generated, of which only four are of interest, with a phylogenetic distribution possibly related to cilia or multiciliation. Cluster 5 contains genes absent in non-ciliated fungi and plants as well as in nematodes, which are also non-ciliated. Cluster 7 contains genes present mainly in Metazoa apart from nematodes as well as several genes known to be involved in multiciliation (*CEP152, PLK4, CCP110*) and cluster 8 contains genes present in chordates, but more interestingly, some of the major genes of multiciliation (*GMNC, CCNO, MCIDAS, CEP63*). Finally, cluster 9 contains genes present in vertebrates but absent in Otomorpha fish, such as *CDC20B*. In total, those 4 clusters contain 514 genes, of which 87 come from our target list of 122 genes, including 76 that have no known role in multiciliation (Supplementary Table S1). 

#### 3.3.3. Identification of Most Promising Candidates

Taken together, the results of the various analyses we performed allow us to highlight several candidate genes of unknown function that seem most likely to be involved in multiciliation: *C1orf189* (chromosome 1 open reading frame 189)*, C20orf85* (chromosome 20 open reading frame 85)*, C5orf24* (chromosome 5 open reading frame 24)*, KIAA1841, FAM181A* (family with sequence similarity 181 member 1), *IQCK* (IQ domain-containing protein K)*, LRRC43* (leucine-rich repeat-containing 43)*, DYDC1* (DPY30 domain-containing protein 1)*, CFAP47* (cilia and flagella associated protein 47)*, ANKRD60* (ankyrin repeat domain-containing protein 60) and *TEX43* (testis expressed protein 43) (Table 3). 

The 11 genes we selected all show a clear lack of characterization: we indeed noted a general absence of GO annotations in their human orthologs, as well as missing information regarding their protein interaction leading to their absence in our STRING analysis network. These genes all belong to phylogenetic profile clusters that could be linked to cilia or multiciliation, with the majority in clusters 8 or 9, both of which contain multiple known genes involved in multiciliation (*CCNO, GMNC, MCIDAS, CDC20B, DEUP1, CCDC78*, etc.). Five of those genes, *C1orf189, FAM181A, IQCK, DYDC1* and *CFAP47,* are of particular interest, as they were found in the ‘multiciliated clusters’ of our transcriptomics analysis. As a reminder, those clusters contain genes influenced by the most factors, including the main multiciliated genes (*MCIDAS, DEUP1, CEP152, PLk4, MYB, CDC20B, FOXJ1, FOXN4, CCNO*, etc.). Interestingly, all candidate genes, with the exception of C5orf24, have been localized to either ciliated cells, photoreceptors or testis in the Human Protein Atlas [63]. Moreover, C20orf85 has been linked to lung cancer [64] and DYDC1 seems to be involved in acrosome biogenesis [65], although IQCK has recently been highlighted in several studies regarding Alzheimer’s disease [66,67]. Combining all this information led us to believe these genes to be particularly interesting new targets for future in-depth studies.

## 4. Discussion

Multiciliogenesis is a complex process involving numerous actors and our current knowledge about its mechanisms still leave a lot of gray areas that we need to shed light upon as shown by the existence of only eight genes associated with multiciliation in the GO database. Our goal was to identify new multiciliated genes to expand our understanding, using several genomics methods and combining them. 

In this study, we have done a large-scale screening of genes using 10 transcriptomics datasets dedicated to the analysis of multiciliation, which, to our knowledge, has never been done before. This allowed us to identify 1873 genes that are overexpressed in multiciliated conditions in at least two different experiments, including 148 with no GO annotations in humans, but also raised an important and frequent problem in the study of multiciliation, which is its tight link to ciliogenesis and the difficulty to separate both processes. It led us to the use of an original comparative genomics approach as an additional criterion to distinguish between multiciliogenesis and ciliogenesis. 

We analyzed a selection of 10 genes known to be involved in multiciliation under the light of evolution, which not only allowed us to add clarity and detail to the current knowledge but also showed the existence of a particular evolutionary pattern linked to multiciliation. Our analysis of the ‘multiciliated locus’ containing *MCIDAS*, *CCNO* and *CDC20B* showed that, while it is not maintained in Otomorpha fish, it is found in other Tetrapods, *L. chalumnae* and *C. milii*, suggesting that it might have been present in the beginning of vertebrate evolution. The rupture of the synteny block in Otomorpha as well as the absence of *CDC20B* shows the existence of a likely complex evolutionary history in these species. A possible consequence of those variations could be that, in Otomorpha, MCIDAS has become facultative for the development of MCCs in some organs, as shown recently by Zhou et al. [60]. Another probable impact of sequence divergence of MCIDAS and CCNO in those species is the apparent reduction of cilia on the surface of their MCCs; observations count less than 16 cilia in pronephric ducts of *D. rerio* [5,68], as opposed to the several hundred generally seen in most other species. 

The marked variations in the protein sequences of MCIDAS and CCNO observed in Otomorpha as well as the absence of CDC20B led us to search for other genes that would follow the same evolutionary patterns. The signature that we associated with multiciliation is very subtle, much more so than that of cilia, for which a classical profiling method based on gene presence and absence leads to excellent results [69]. This led us to the development of a new and finer strategy. We used BLUR, a program specifically designed to highlight protein and sub-protein variations between two groups of species to compare Otomorpha and Euteleosteomorpha, another group of bony fishes, which showed that, besides multiciliation, several processes diverged between these two clades, such as immunity. This showed the relevance of combining the two approaches, which highlighted a list of 114 genes that were both functionally linked to multiciliation but also presented a specific evolutionary pattern. 

The functional characterization of these genes using the STRING database confirmed that a large part of our targets interacted with known ciliary genes, but also highlighted one common problem of in silico studies, which is the shortage of data and annotations in some cases, as shown by the lack of functional interactions detected for 65 of our target genes. This led us to the analysis of the phylogenetic profiles of our target genes, with two thirds of our target list showing a distribution that correlates to either a ciliary or a multiciliary pattern. 

In this study, we highlighted a list of 114 potentially multiciliated target genes, of which 11 were especially promising: *C1orf189, C20orf85, C5orf24, KIAA1841, FAM181A IQCK, LRRC43, DYDC1, CFAP47, ANKRD60* and *TEX43*. Highlighted in our various analysis as either presenting an evolutionary signature that correlates with multiciliation, with a divergence in Otomorpha and a distribution in ciliated species, or as heavily influenced by the main transcription factors of the multiciliated pathway, these genes are, as of today, poorly characterized. With the exception of DYDC1, they are defined as TDARK according to the system developed by the Illuminating the Druggable Genome Knowledge Management Center (IDG-KMC), i.e., they correspond to targets about which virtually nothing is known. Their lack of annotations makes them perfect targets for further studies and particularly encouraging candidates for multiciliation. Experimental validation is now required for these results, which could not only improve our understanding of the complex process that is multiciliation but could also be helpful as part of variant analyses in pathologies linked to multiciliation, for which very few responsible genes are currently known.

## Figures and Tables

**Table 1:** Overview of the different datasets and experiments used for the functional analysis.

GEO Accession	Overall Experimental Design (Multiciliation vs. No Multiciliation)	Species	Experiment Type
GSE32452 [19]	Notch intracellular domain (ICD) + glucocorticoid inducible Multicilin vs. ICD	*Xenopus laevis*	Microarray
GSE59309 [23]	Inducible Multicilin vs. inducible Multicilin + truncated E2F4	*X. laevis*	RNASeq
GSE89271 [38]	Inducible Multicilin vs. inducible Multicilin + Foxn4 morpholino	*X. laevis*	RNASeq
	Inducible Multicilin vs. inducible Multicilin + CRISPR/Cas9 Foxn4 mutant	*X. laevis*	RNASeq
	Inducible Multicilin vs. inducible Multicilin + CRISPR/Cas9 Foxj1 mutant	*X. laevis*	RNASeq
GSE76342 [39]	Notch- vs. ICD; ICD vs. ICD + Multicilin; Notch- vs. Notch- + Multicilin-	*X. laevis*	RNASeq
GSE60365 [40]	Non-targeted shRNA vs. Myb shRNA	*Mus musculus*	Microarray
GSE75715 [25]	Wild Type vs. p73 knockout	*M. musculus*	RNASeq
GSE73331 [41]	Wild Type vs. E2F4 knockout	*M. musculus*	Microarray
GSE116690 [18]	Stk11+ vs. Stk11-	*M. musculus*	RNASeq

**Table 2:** Cluster regrouping according to similarity in profiles. Pertinent GO terms were selected among the ‘biological process’ GO annotation dataset and the results with the lowest *p*-value.

Clusters	Characteristics	Genes	GO Terms	*p*-Value
3, 6, 7, 14	Foxj1/Foxn4 targets	759	‘establishment of localization’	1.64 × 10^−13^
17, 18, 19, 26, 27	MCIDAS/E2F4 complex and Foxj1 or Foxn4 targets	308	‘cilium organization’	8.28 × 10^−23^
22, 24, 25, 28	Mouse genes	160	‘cilium movement’	1.32 × 10^−9^
20	MCIDAS/E2F4 complex targets	58	‘centrosome cycle’	1.30 × 10^−13^
8, 9, 12, 13	Multiciliated clusters	587	‘cilium organization’	1.94 × 10^−92^

**Table 3:** Most promising target genes highlighted by all methods.

Gene	Transcriptomics Cluster	BLUR	STRING Cluster	Profiling Cluster
*C1orf189*	13	Absent Otomorpha	-	9
*C20orf85*	18	Absent Otomorpha	-	9
*C5orf24*	3	Mildly likely divergence	-	8
*KIAA1841*	19	Mildly likely divergence	-	5
*FAM181A*	9	Highly likely divergence	-	8
*IQCK*	12	Mildly likely divergence	-	7
*LRRC43*	22	Mildly likely divergence	-	8
*DYDC1*	8	Mildly likely divergence	-	8
*CFAP47*	12	Absent Otomorpha	-	5
*ANKRD60*	17	Absent Otomorpha	-	9
*TEX43*	18	Absent Otomorpha	-	9

## Data Availability

Publicly available datasets were analyzed in this study. These data can be found in the GEO database (datasets GSE32452, GSE59309, GSE89271, GSE76342, GSE60365, GSE75715, GSE73331, GSE116690).

## Supplementary Materials

The following are available online at https://www.mdpi.com/article/10.3390/genes12091452/s1, Table S1: List of target genes identified by the combined functional and comparative genomics approaches characterized using protein interaction annotations and phylogenetic profiling.
